# Supplementary material for: Effect of Pefloxacin on Clostridioides difficile R20291 Persister Cells Formation
Source: Antibiotics (Basel). 2025 Jun 20;14(7):628. doi: 10.3390/antibiotics14070628 (PMC12291809; doi:10.3390/antibiotics14070628)
Supplement: Supplementary file 1 [file antibiotics-14-00628-s001.zip › antibiotics-3672159-supplementary.pdf]

Table S1: Primers used in this study

Primer	Sequence (5′-3′)
**qPCR_clpP1_F**	TATGGGAGCATTCTTGTTGGCA
**qPCR_clpP1_R**	AATGGCTGACCAGTTCTTTCAG
**qPCR_clpP2_F**	CCCTGGTGGCTCTGCTACATCA
**qPCR_clpP2_R**	CCTCCCATTGGCTGATGAATCA
**qPCR_LON_F**	ATGAAGGAATCTGCGAAGACAGG
**qPCR_LON_R**	TGCTGAGATTACTGCAAGAGCCA
**qPCR_clpB_F**	CCCAGAAGGCACATATGATGCA
**qPCR_clpB_R**	CCTACACCTGGCTCACCAATCA
**qPCR_clpC_F**	TTACACGGGCAACCAGAATCAA
**qPCR_clpC_R**	TGGCTGTTTTCCCTACACCTGG
**qPCR_clpX_F**	TGCAATGGCAGATGCTACATCA
**qPCR_clpX_R**	TGGGTTTTCAGATTTTCTTGCA
**qPCR_mazF_F**	CGCTGATTTAAGTCCAGTTGTTGG
**qPCR_mazF_R**	GCCCATATTCATTGGAACTTATCT
**qPCR_relE_F**	TACAAACGAGCCATCAAGCGTG
**qPCR_relE_R**	GTCCGGGAGAATGTGACATTCC
**qPCR_recA_F**	TAGCAGCATTAGTTCCAAAGGC
**qPCR_recA_R**	GTGCACGTCCCTCCAGTAGTAG
**qPCR_dnaF_F**	TCCATCTATTGCAGGGTGGT
**qPCR_dnaF_R**	CCCAACTCTTCGCTAAGCAC
**qPCR_16s_F**	GGGAGACTTGAGTGCAGGAG
**qPCR_16s_R**	GTGCCTCAGCGTCAGTTACAGT

Table S2: Differential slope changes between time-kill persister curves for 50× MIC and 100× MIC of pefloxacin

Slope/MIC	50× MIC	100× MIC
ΔlogCFU/Δtime fase 1 (0-5h)	-0,65	-0,40
ΔlogCFU/Δtime fase 2 (5-8h)	-0,06	-0,55

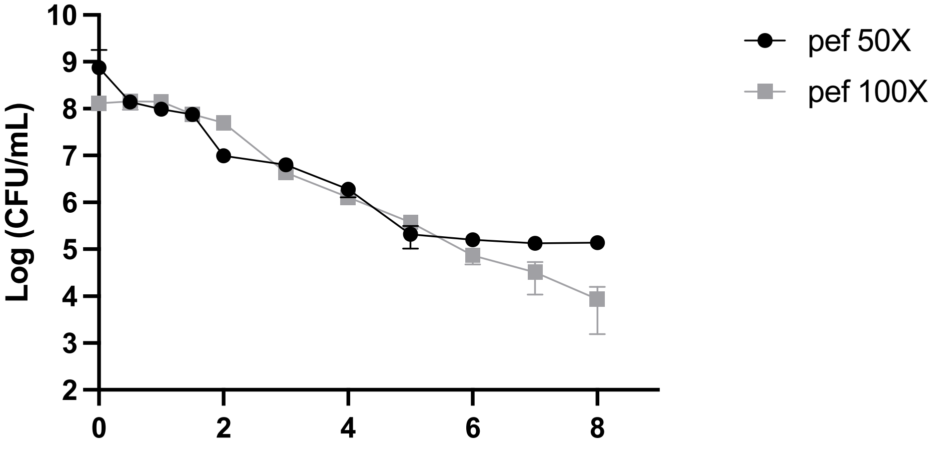

Figure S1: Pefloxacin 50× MIC and 100× MIC effect on persistence in *C. difficile* R20291. Time–kill kinetics of exponential-phase cultures treated with pefloxacin at 50× and 100× the determined MIC (1.53 μg/mL). 50× display a characteristic biphasic killing curve: an initial rapid decline in viable counts followed by a persistent plateau (*n* = 3; mean ± SD).

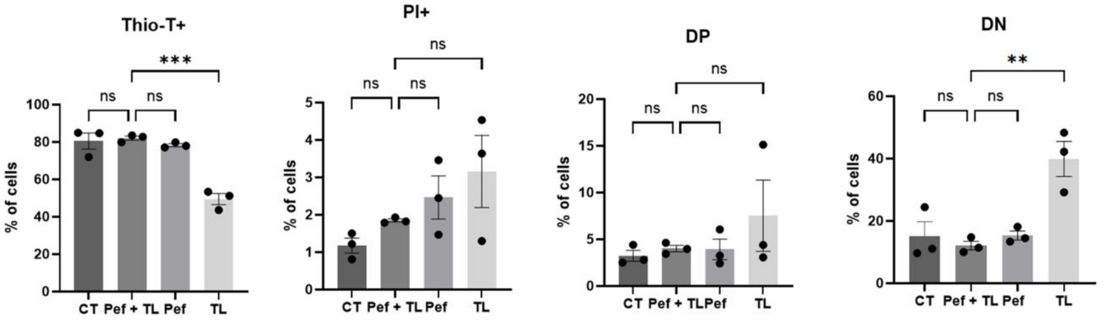

Figure S2: Percentage of cells labeled with thioflavin-T (Thio-T), propidium iodide (PI), both stained (double positive; DP) or unlabeled (double negative; DN) (n=3). Error bars represent standard error (*** $p < 0.001$; ** $p < 0.005$; ns= not significant).

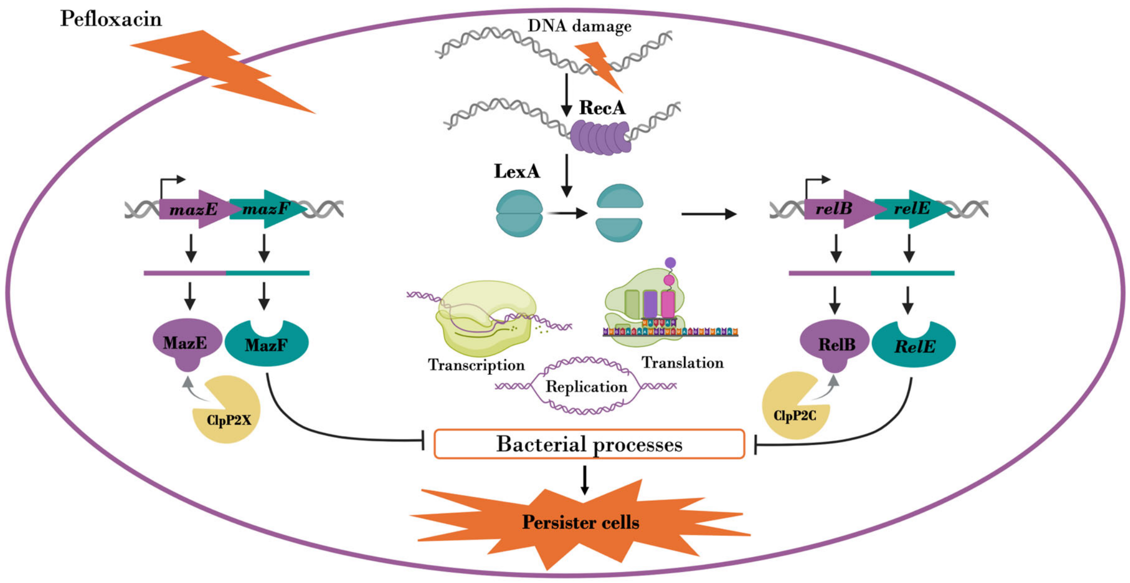

Figure S3: Model representing the possible activation of TA and SOS systems in *Clostridioides difficile* persister cells formation under pefloxacin treatment
